# Supplementary material for: Thecamoebians (Testate Amoebae) Straddling the Permian-Triassic Boundary in the Guryul Ravine Section, India: Evolutionary and Palaeoecological Implications
Source: PLoS One. 2015 Aug 19;10(8):e0135593. doi: 10.1371/journal.pone.0135593 (PMC4546057; doi:10.1371/journal.pone.0135593)
Supplement: S2 Fig — (DOC) [file pone.0135593.s002.doc]

**Thecamoebians (Testate Amoebae) straddling the Permian-Triassic Boundary in the Guryul Ravine Section, India: Evolutionary and palaeoecological implications**

Vartika Singh^1^, Sundeep K. Pandita^2^, Rajni Tewari^1,*^, Peter J. van Hengstum^3^, Suresh S. K. Pillai^1^, Deepa Agnihotri^1^, Kamlesh Kumar^1^, G. D. Bhat^4^

1. Birbal Sahni Institute of Palaeobotany, 53 University Road, Lucknow, 226007, India

2. Department of Geology, University of Jammu, Jammu, 180006, India

3. Department of Marine Sciences, Texas A&M University at Galveston, Galveston, Texas, 77553, USA

4. Directorate of Geology and Mining, Jammu and Kashmir Government, Srinagar, 190002, India

* Corresponding author: rajni_tewari@bsip.res.in

**Abstract**

Exceptionally well-preserved organic remains of thecamoebians (testate amoebae) were preserved in marine sediments that straddle the greatest extinction event in the Phanerozoic: the Permian-Triassic Boundary. Outcrops from the Late Permian Zewan Formation and the Early Triassic Khunamuh Formation are represented by a complete sedimentary sequence at the Guryul Ravine Section in Kashmir, India, which is an archetypal Permian-Triassic boundary sequence [1]. Previous biostratigraphic analysis provides chronological control for the section, and a perspective of faunal turnover in the brachiopods, ammonoids, bivalves, conodonts, gastropods and foraminifera. Thecamoebians were concentrated from bulk sediments using palynological procedures, which isolated the organic constituents of preserved thecamoebian tests. The recovered individuals demonstrate exceptional similarity to the modern thecamoebian families Centropyxidae, Arcellidae, Hyalospheniidae and Trigonopyxidae, however, the vast majority belong to the Centropyxidae. This study further confirms the morphologic stability of the thecamoebian lineages through the Phanerozoic, and most importantly, their apparent little response to an infamous biological crisis in Earth’s history.

**Introduction**

The Permian-Triassic Boundary (PTB) extinction was a catastrophic event in Earth’s history, where more than 90% of marine and 70% terrestrial life went extinct [2, 3]. Multiple studies have examined the timing, nature and biogeographic extent of this extinction event, including the low to middle palaeo-latitude sites in the Palaeotethys [4-13], part of Panthalassa around Japan [14], and in the northern high latitudes [15-20]. However, the high southern palaeolatitude PTB successions have become a cynosure to study the extinction patterns of marine and non-marine fauna [19, 21-27]. The Guryul Ravine PTB succession in the Kashmir Northwest Himalaya belonged to the peri-Gondwanan region that covered the northern margin of Gondwana and the southern margin of Palaeotethys/Neo-Tethys (S1 Fig.1). Well-preserved marine [28-35] elements have been recovered from this section, as well as some terrestrial [36] remains, which provide an important biologic perspective of the PTB event (Fig. 2).

Thecamoebians (testate amoebae) are eukaryotic heterotrophic protists that are polyphyletic in origin, based on molecular RNA analysis [37]. In modern environments, they colonize fresh to slightly brackish water aquatic environments (<4 psu) [38] and they form a simple secreted (autogenous) or agglutinated (xenogenous) test (or shell) that can be preserved in the sedimentary record following their death. Owing to their tight ecological zonation with respect to salinity, pH, and moisture content, subfossil thecamoebian assemblages preserved in late Holocene successions are used to reconstruct salinity [39, 40] and sea-level variations [41,42], precipitation variability [43] and anthropogenic impacts on coastal and lacustrine environments [44,45]. However, their pre-Quaternary fossil record is sparse, promoting some uncertainty in our understanding on the evolution of important taxonomic lineages, and the group’s response to critical environmental perturbations in Earth’s history.

The thecamoebian fossil record is currently dominated by Mesozoic observations [46-54], with few in the Paleozoic and Tertiary [55-64] (S2 Fig. 3). The preservation of the thecamoebians is like other organic-based microfossil groups, where agglutinated test have a better preservation potential than more fragile autogenous test [46]. Some of the better-preserved fossil thecamoebian assemblages have been recovered from both lacustrine and eustuarine successions that are nearly identical to modern forms [46,55,65,66], which suggests little morphologic variability in the group over the Phanerozoic [47-49,50,56,57, 65-69] (S2 Fig. 3). The oldest fossil record of the thecamoebians is from the Neoproterozoic [65,70] with accounts also available from the Carboniferous [71]. Early Permian thecamoebians from the Tethys Himalaya are presented by Kumar et al. [66], and Late Permian thecamoebians have recently been recorded from the Raniganj Formation of the Godavari Graben [68]. Thus far, the thecamoebian fossil record is represented by eleven modern families: Arcellidae, Centropyxidae, Plagiopyxidae, Difflugidae, Hyalophenidae, Phyrganellidae, Euglyphidae, Cyphoderiidae, Amphitremidae, Trigonopyxidae and Trinematidae [46, 66, 69].

This paper presents the first account of the thecamoebians straddling the PT Boundary at the Guryul Ravine PTB Section in India, and the only one record of Late Permian thecamoebians from the Northwest Himalayan region of Jammu and Kashmir. These results provide direct evidence for the successful crossing of the thecamoebian (testate amoebae) group across the PT Boundary extinction event.

**Geological Setting**

The Guryul Ravine Section lies in the northernmost Indian state of Kashmir [72] (Fig. 4), the geology of which has been extensively reviewed by by Nakazawa et al. [27], Kapoor [1] and Tewari et al [36]. During the Late Permian and Early Triassic, the Kashmir region of the Indian sub-continent was located in northern Gondwana, at 35°S palaeolatitude along the southern margin of the Tethys Sea [73,74]. The depositional setting of the Late Permian Zewan Formation was a shallow marine environment with relatively high terrigenous sediment supply, whereas the Early Triassic Khunamuh Formation was deposited during a transgressive episode [74]. Marine sediments of the Guryul Ravine accumulated above pre-existing volcanic rocks [1]. Isotopic evidence of Proemse et al., 2013 [75] indicates relatively oxic conditions in the shallow marine regions of the Northwest margin of Pangea throughout the Late Permian Mass Extinction (LPME). However, the organic carbon flux study of Algeo et al., 2013 [76], and others [74,77] on the PTB sections have suggested that the well-oxygenated conditions were briefly interrupted by periods of anoxia in the Late Permian-Early Triassic shallow marine environments. The Guryul Ravine Section represents a continuous gradational sequence across the PT Boundary, whereas depositional hiatuses present in other Kumaon and Spiti Himalayas sections preclude a continuous record of paleoenvironmental changes during the PT Boundary Event. Our study of organic matter at the Guryul Section [36], reveals the prevalence of amorphous organic matter (AOM), which perhaps suggests a regional prevalence of anoxic conditions.

Stratigraphically, both the Zewan and Khunmuh Formations have been further sub-divided into members and units, respectively, based on lithological and paleontological characteristics. The Zewan Formation is divided into four Members (A-D) [1] (Fig. 2). Carbonate rocks and sandy shale comprise Member A, Member B is shale with low carbonate content, Member C is thick bedded sandy limestone, sandy shale and muddy sandstone, and Member D is calcareous muddy sandstone. Only Members A, C and D are rich in marine fossils. An abrupt change in sedimentation to intercalated grey to black limestone and black shale demarcates the onset of the Khunamuh Formation. This Formation is divided in to six members E-J (Fig. 2), with the lower units in Member E marking the PT Boundary Event. Member E is sub-divided into Units E_1_, E_2_ and E_3_. The Unit E1 contains mixed faunal elements of Late Permian and Early Triassic age. The PT Boundary has been placed at the base of the conodont *Hindeodus parvus* zone at the contact of E_1_ and E_2_ (Fig. 2).

Pioneering faunal work at the Guryul Ravine PTB Section was carried out by [28], who identified four faunal divisions: I to III in the Zewan Formation (Late Permian) and IV in the Unit E1 of the Khunamuh Formation (Early Triassic) (Fig. 2). The faunal divisions I and II correspond to the units A and B, respectively, and contain bryozoans, brachiopods and foraminifers. The faunal diversity of division II is less than that of division I. The faunal division III is displayed by the litho units C and D and shows the dominance of gastropods and bivalves over brachiopods. The faunal division IV is displayed by bivalves and ammonoids. Brachiopods, bivalves and conodonts have also been recovered from this faunal division, which provide chronological control for the section (Fig. 2).

**Material and Methods**

This study is a part of Birbal Sahni Institute of Palaeobotany (BSIP) Project Number 2.3 entitled “Mega- and microfloristics of the Permo-Carboniferous sediments of Kashmir region: Evolutionary, biostratigraphical, palaeoecological and palaeophytogeographical implications” under Thrust Area 2— “Phanerozoic Terrestrial and Coastal Ecosystems: Biostratigraphical, Palaeoenvironmental, Palaeoecological And Palaeobiogeographical Aspects”. All necessary permits were obtained from the Director, BSIP for the field visit and the described study, which comply with all relevant regulations.

A total of nine bulk sediment samples (GR1 to GR9) were collected from C and D members of Zewan Formation and E Member of Khunamuh Formation (Fig. 2), which were processedfollowing the palynological procedure used by Kumar et al, [66]. Samples were first treated with 30% hydrochloric acid followed by wet sieving on a 20 µm-mesh to concentrate microfossils, with the recovered residue mounted on slides with canada balsam. Prepared slides were then studied under a high power light Microscope Leitz Laborlux D to study morphological features of the recovered thecamoebian tests.

The absolute abundance of the number of recovered forms of different species has also been studied (Fig. 5). Morphological data of all the examined individuals has been given in tabular form. (Table 1). The slides are deposited in the repository of the Birbal Sahni Institute of Palaeobotany, Lucknow (www.bsip.res.in/Museum.html) vide museum statement no. 1354. The museum accession numbers of the slides are 14869, 14971-14976 and 14881. Existing taxonomic guides from Medioli *et al.* [78-80], Smirnov *et al.* [81] and Ogden and Hedley [82] were followed for thecamoebian identification.

**Results and Systematics**

A total of 24 thecamoebians were recovered from the Guryul Ravine Section, most of which can be confidently assigned to the modern genera *Centropyxis*, *Arcella*, *Cucurbitella*, and *Cyclopyxis*. From the Zewan Formation in the Late Permian (C Member, Sample GR-2 on Fig. 2), *Centropyxis* *aculeata*, *C. aculeata* var. *spinosa* and *Centropyxis aculeata* var. *discoides* were recovered. The *Centropyxis aculeata* tests preserved in the Member C show very less Brown Degraded Organic Matter (BDOM). In the Early Triassic Khunamuh Formation, thecamoebians were recovered from Member E in the Units E2 (Sample GR-8) and E3 (Sample GR-9). In E2, *Centropyxis aculeata*, *Centropyxis aculeata* var. *spinosa*, *Centropyxis aculeata* var. *discoides*, forms belonging to *Centropyxis constricta*-complex, Centropyxis Stein 1857, *Centropyxis platystoma*, *Cucurbitella tricuspis*, *Cyclopyxis eurystoma* and an unidentified individual from Unit E_2_. *Arcella* and centropyxids were recovered from the Unit E_3_ Unit of the Khunamuh Formation. The thecamoebian forms preserved in the Unit E2 and E3 show fairly large amount of BDOM. Similarly, our sedimentary dispersed organic matter study of the GR section shows very low BDOM content in the Member C as compared to the Units E2 and E3 having relatively high BDOM [36]. In the Early Triassic Khunamuh Formation, occurrence of thecamoebians in the Unit E_3_ is relatively low in comparison to the Member C and Unit E_2_. One form that appears to have two apertures remains unidentified (incertae sedis) (Fig. 6: K, L), which may or may not be an artifact of taphonomic processes. The recovered thecamoebians can be organized into the following systematics:

Phylum Amoebozoa [83]

Subphylum Lobosa [84,85]

Class Tubulinea [86,87]

Order Arcellinida [88]

Family Arcellidae [89]

Genus *Arcella* [89] (Fig. 6: J)

Family Centropyxidae [90]

Genus *Centropyxis* [91]

*Centropyxis* [91] (Fig. 6: D)

*Centropyxis aculeata* [92] (Fig. 8: C-L)

*Centropyxis aerophila*-complex [93] (Fig. 6: E-G)

*Centropyxis aculeata var. discoides* [94] (Fig. 7: G-L)

*Centropyxis aculeata var. spinosa* (Fig. 7: A-F; Fig. 8: A, B)

Family Hyalospheniidae [95]

Genus Cucurbitella, [96]

*Cucurbitella tricuspis* [97] (Fig. 6: C)

Family Trigonopyxidae [98]

Genus *Cyclopyxis*

*Cyclopyxis eurystoma* [98] (Fig. 6: H, I)

**Discussion**

Despite the overall low recovery of fossil thecamoebians from the Guryul Ravine Section, their recovery at such a critical environmental transition has significant taxonomic and ecological implications. Many of the preserved individuals are intact organic linings as a result of the palynological preparation procedures, and like individuals preserved in Mesozoic amber, allows for an examination of the autogenous (or secreted) mucopolysaccharide test structure. In contrast, other fossil thecamoebians that are typically sieved from bulk sediments, preferentially congregate individuals with agglutinated (or xenogenous) test and damaging many of the organic structures. Structures like inner shell lining, apertural bridges, and also test ornamentation (i.e., spines on the fundus) are all evidenced in the collection from the Guryul Ravine Section (Figs. 6-8). In addition, pores around the periphery of the aperture in the autogenous test of *Arcella* are not commonly preserved in fossil Paleozoic thecamoebians. However, they have been documented in Permian examples by Kumar et al. [66] and Farooqui et al. [68] (Fig. 7: J). Such test characteristics are easily observed in the modern thecamoebian lineages and yet rarely observed in fossil examples.

The most significant result of this study is providing direct evidence that very common thecamoebian genera successfully crossed the PT Boundary (e.g., *Centropyxis* and *Arcella*). Previously, this could be inferred based on their preservation in both Late Paleozoic and Mesozoic amber deposits, but the Guryul Ravine Section provides individuals on other side of the boundary at the same paleogeographic locale. These observations attest to the resiliency of the thecamoebian group to global climatic events through the Phanerozoic.

An important omission in this collection is the lack of *Difflugia* preserved in the Guryul Ravine Section. Many specimens comprise the genus *Difflugia,* which are abundant in late Holocene freshwater environments. It is possible that taphonomic issues prevented their preservation, but this seems unlikely given the preservation of more fragile autogenous *Arcella* tests. In other fossil thecamoebian collections that have been sieved out of bulk sedimentary samples, individuals of *Difflugia* have been more common than *Centropyxis* [46]. It is possible that this generic bias is related to sample processing techniques (sieving vs. chemical palynological processing).

In general, there are very few studies on thecamoebians from deep-time sequences. The earliest record of thecamoebians is likely from Neoproterozoic marine sediments of Chuar group, Grand Canyon, which were originally described as Vase Shaped Microfossils (VSM’s) [65]. Since modern thecamoebians are not found in marine settings, this perhaps suggests a marine origin for the group with their ecological shift to lacutrine or brackish environments in the Early to Middle Paleozoic. During the Carboniferous when land plants greatly diversified and formed widespread swamp depositional environments, thecamoebians were preserved in the resultant organic-rich coal deposits now presently located in Nova Scotia, Canada [99, 69, 56]. The recovered fossil thecamoebians from the PT Boundary at the Guryul Ravine Section can be interpreted in one of two ways. The thecamoebians were transported to the locality along with other terrigenous material from the adjacent coastal zone, or perhaps they represent an in situ or primary assemblage. The implications of the later interpretation would be that some members of the thecamoebian group also occupied marine habitats in some capacity through the entirety of the Paleozoic. For example, the two Late Permian occurrences of *Centropyxis*, one from the fresh water sediments of the Raniganj Formation, Godavari Graben [68] and the other from the marine sediments of Zewan Formation (this study) suggest that the genus occupied both fresh water as well as marine environments during this time. Similarly, the genus *Arcella* has been reported from Carboniferous freshwater coals [56] and from the Late Permian sediments of Raniganj Formation at Lingala-Koyagudam coal belt, Godavari graben [68], whereas this study presents the occurrence of *Arcella* from the marine Early Triassic Unit E3. If indeed thecamoebians occupied marine environments throughout the Paleozoic, however, it remains puzzling why more occurrences of marine thecamoebians have not been reported in the global palynological surveys of Paleozoic marine sediments. Therefore, it remains important to continue documenting fossil thecamoebians to accurately resolve their Paleozoic paleoecological tolerance to salinity and evolutionary history.

Another important observation based on this PT Boundary collection is the variability in the absolute number of spines ornamented onto the tests in the species *Centropyxis.* The taxonomy of these species remains complex, in part because of the intragradational character in the external morphology of many thecamoebian species [100,101]. Some authors consider dividing this genus into species based on the absolute number of spines ornamenting the test or their geometric orientation [102,103]. Most thecamoebian workers agree with the notion of considerable phentoypic plasticity in the *Centropyxis* genus, and recognize that the most important variability for paleoecologic work is taxonomic consistency. Here, the recovered centropyxids are also phenotypically diverse, with multiple numbers of spines that we divide into morphotypes, or variants, based on the absolute number of spines. For example, the diagnosis of *Centropyxis aculeata* var. *spinosa* includes an individual with >3 spines ornamenting the posterior of the test [103]. The ability to discern such taxonomic detail is remarkable given the age of the specimens, and indicates that phenotypic plasticity in the number of spines is a long-lived characteristic in this genus. Even in modern thecamoebians, however, the functional ecology of this test ornamentation remains unknown.

**Conclusions**

This study documents exceptionally well-preserved thecamoebians across the PT Boundary event from the Guryul Ravine Section in Kashmir, India. The site was paleogeographically situated at the northern margin of Gondwana, and the recovered thecamoebians are from the Late Permian Zewan and the Early Triassic Khunamuh Formations. The Guryul Ravine Section is an archetypal PT Boundary sequence, and the thecamoebians provide an additional faunal signature to the previously documented foraminifera, brachiopods, bivalves, ammonoids and conodonts. The recovery and preservation of the thecamoebians from the Guryul Ravine Section is not only new to this section but for any PTB section worldwide. The preservation of these fragile microfossils was likely enhanced by punctuated local or regional anoxic/dysoxic conditions, which are evinced by the presence of amorphous organic matter in this section [36]. Even the intraspecific variability within the *Centropyxis* genera could be observed in the recovered individuals. Thecamoebians have a poorly resolved fossil record, but the results presented here confidently indicate that some of the most common modern thecamoebian genera successfully transitioned across the PT Boundary extinction event with apparently little ecologic challenge.

**Acknowledgments**

We thank Professor Sunil Bajpai, Director of the Birbal Sahni Institute of Palaeobotany in Lucknow for providing necessary facilities to carry out the research, discussions with Alexey Smirnov on taxonomy, and to the anonymous reviewers for providing constructive suggestions. This is BSIP contribution no. 66/2013-14.

**References**

1. Kapoor HM. The Guryul ravine section, candidate of the global stratotype and point (GSSP) of the Permian-Triassic boundary (PTB). Yin H, editor. The Paleozoic-Mesozoic Boundary: Candidates of the Global Stratotype Section and Point of the Permian-Triassic. 1996.
2. Erwin DH. The Great Paleozoic Crisis: Life and Death in the Permian. Columbia University Press, New York; 1993. 327 pp.
3. Erwin DH. The Permo-Triassic extinction. Nature. 1994; 367: 231-236.
4. Wignall PB, Hallam A. Anoxia as a cause of the Permian/Triassic mass extinction: facies evidence from northern Italy and the western United States. Palaeogeogr Palaeoclimatol Palaeoecol. 1992; 93: 21-46.
5. Bowring SA, Erwin DH, Jin YG, Martin MW, Davidek K, Wang W. U/Pb zircon geochronology and tempo of the end- Permian mass extinction. Science. 1998; 280: 1039-1045.
6. Jin YG, Wang Y, Wang W, Shang QH, Cao CQ, Erwin DH. Pattern of marine mass extinction near the Permian-Triassic boundary in South China. Science. 2000; 289: 432-436.
7. Kaiho K, Kajiwara Y, Nakano T, Miura Y, Kawahata H, Tazaki K. et al. End-Permian catastrophe by a bolide impact: evidence of a gigantic release of sulphur from mantle. Geology. 2001; 29: 815-818.
8. Krull ES, Lehrmann DJ, Druke D, Kessel B, Yu YY, Li R. Stable carbon isotope stratigraphy across the Permian- Triassic boundary in shallow marine carbonate platforms, Nanpanjiang Basin, south China. Palaeogeogr Palaeoclimatol Palaeoecol. 2004; 204: 297-315.
9. Mundil R, Metcalfe I, Ludwig KR, Renne PR, Oberli F, Nicoll R.S. Timing of the Permian-Triassic biotic crisis: implications from new zircon U/Pb age data (and their implications). Earth Planet. Sci. Lett. 2001; 187: 131-145.
10. Mundil R, Ludwig KR, Metcalfe I, Renne PR. Age and timing of the Permian mass extinctions: U/Pb dating of closedsystem zircons. Science. 2004; 305: 1760-1763.
11. Shen SZ, Crowley JL, Wang Y, Bowring SA, Erwin DH, Sadler PM, et al. Calibrating the End-Permian Mass Extinction. Science. 2011; 334, 1367-1372.
12. Song H, Wignall PB, Tong J, Yin H. Two pulses of extinction during the Permian–Triassic crisis. Nat. Geosci. 2013; 6: 52-56.
13. Burgess SD, Bowring S, Shen SZ. High-precision timeline for Earth’s most severe extinction. Proc. Natl. Acad. Sci. 2014; 111: 1-6.
14. Isozaki Y. Permo-Triassic boundary superanoxia and stratified superocean: records from lost deep-sea. Science. 1997; 276: 235-238.
15. Henderson CM. Uppermost Permian conodonts and the Permian-Triassic boundary in the western Canada sedimentary basin. Bull. Can. Petro. Geol. 1997; 45(4): 693-707.
16. Henderson CM, Baud A. Correlation of the Permian-Triassic boundary in Arctic Canada and comparison with Meishan, China. In: Wang NW, Remane J, editors. Proceedings of the 30th International Geological Congress. 1997, 11: 143-152.
17. Wang K, Geldsetzer HHJ, Krouse HR. Permian-Triassic extinction: organic ^13^C evidence from British Columbia, Canada. Geology. 1994; 22: 580-584.
18. Wignall PB, Morante R, Newton R. The Permo-Triassic transition in Spitsbergen: 13Corg chemostratigraphy, Fe and S geochemistry, facies, fauna and trace fossils. Geol. Mag. 1998; 135: 47-62.
19. Wignall PB, Newton R. Contrasting deep-water records from the Upper Permian and Lower Triassic of south Tibet and British Columbia: evidence for a diachronous mass extinction. Palaios. 2003; 18: 153-167.
20. Twitchett RJ, Looy CV, Morante R, Visscher H, Wignall PB. Rapid and synchronous collapse of marine and terrestrial ecosystems during the end-Permian mass extinction event. Geology. 2001; 29: 351-354.
21. Smith RMH, Ward PD. Pattern of vertebrate extinctions across an event bed at the Permian-Triassic boundary in the Karoo Basin of South Africa. Geology 2001; 29: 1147-1150.
22. Shen SZ, Shi GR. Paleobiogeographical extinction patterns of Permian brachiopods in the Asian-western Pacific Region. Paleobiology. 2002; 28(4): 449-463.
23. Brookfield ME, Twitchett RJ, Goodings C. Palaeoenvironments of the Permian-Triassic transition sections in Kashmir, India. Palaeogeogr Palaeoclimatol Palaeoecol. 2003; 198: 353-371.
24. Krystyn L, Baud A, Richoz S, Witchett RJ. A unique Permian-Triassic boundary section from Oman. Palaeogeogr Palaeoclimatol Palaeoecol. 2003; 191: 329-344.
25. Sarkar A, Yoshioka H, Ebihara M, Naraoka H. Geochemical and organic carbon isotope studies across the continental Permo-Triassic boundary of Raniganj Basin, eastern India. Palaeogeogr Palaeoclimatol Palaeoecol. 2003; 191: 1-14.
26. Retallack GJ, Smith RMH, Ward PD. Vertebrate extinction across Permian-Triassic boundary in Karoo Basin, South Africa. Bull. Geol. Soc. Am. 2003; 115: 1113-1152.
27. Twitchett RJ, Krystyn L, Baud A, Wheeley JR, Ricjoz S. Rapid marine recovery after the end-Permian mass-extinction event in the absence of marine anoxia. Geology. 2004; 32: 805-808.
28. Nakazawa K, Kapoor HM, Ishii K, Bando Y, Okimura Y, Tokuoka T. The Upper Permian and the Lower Triasssic in Kashmir, India. Memoirs of the Faculty of Science, Kyoto University, Series of Geology and Mineralogy. 1975; 41: 1-106.
29. Nakazawa K, Kapoor HM. editors The Upper Permian and Lower Triassic Faunas of Kashmir. Palaeontologia Indica, New Series.1981; 46: 1-191.
30. Matsuda T. Early Triassic Conodonts from Kashmir, India part 1: Hindeodus and Isarcicella. J Geosci. Osaka City University. 1981; 24: 75-108.
31. Matsuda T. Early Triassic Conodonts from Kashmir, India part 2: Neospathodus 1. J Geosci. Osaka City University. 1982; 25: 87-102.
32. Matsuda T. Early Triassic Conodonts from Kashmir, India part 3: Neospathodus 2. J Geosci. Osaka City University. 1983; 26: 87-110.
33. Matsuda T. Early Triassic Conodonts from Kashmir, India part 4: Gondolella and Platyvillosus. J Geosci. Osaka City University. 1984; 27: 119-141.
34. Furnish WM, Glenister BF, Nakazawa K, Kapoor HM. Permian ammonoid Cyclolobus from the Zewan Formation, Guryul Ravine, Kashmir. Science. 1973; 180: 188-190.
35. Korte C, Pande P, Kalia P, Kozur HW, Joachimski MM, Oberhansli H. Massive volcanism at the Permian-Triassic boundary and its impact on the isotopic composition of the ocean and atmosphere. J Asian Earth Sci. 2010; 37: 293-311.
36. Tewari R, Ram-Awatar, Pandita SK, McLoughlin S, Agnihotri D, Pillai SSK, Singh V, Kumar K, Bhat GD. The Permian-Triassic palynological transition in the Guryul Ravine section, Kashmir, India: implications for Tethyan-Gondwanan correlations. Earth-Science Rev. 2014 (In press).
37. Pawlowski J, Burki F. Untangling the phylogeny of amoeboid protists. J Eukar Microbiol. 2009; 56: 16-25.
38. van Hengstum PJ, Reinhardt EG, Beddows PA, Huang RJ, Gabriel JJ. Thecamoebians (testate amoebae) and foraminifera from three anchialine cenotes in Mexico: Low salinity (1.5 - 4.5 psu) faunal transitions. J Foramin Res. 2008; 38: 305-317.
39. Scott DB, Medioli FS, Schafer CT. Monitoring in Coastal Environments Using Foraminifera and Thecamoebians Indicators. 2001.  (Cambridge University Press, 2001).
40. van Hengstum PJ, Reinhardt EG, Beddows PA, Gabriel JJ. Investigating linkages between Holocene paleoclimate and paleohydrogeology preserved in Mexican underwater cave sediments. Quat Sci Rev. 2010; 29: 2788-2798.
41. Charman DJ, Roe HM, Gehrels WR. The use of testate amoebae in studies of sea-level change: a case study from the Taf estuary, South Wales, UK.The Holocene. 1998; 8: 209-218.
42. Gehrels WR, Roe HM, Charman DJ. Foraminifera, testate amoebae and diatoms as sea-level indicators in UK saltmarshes: a quantitative multi-proxy approach. J Quat Sci. 2001; 16: 201-220.
43. Woodland WA, Charman DJ, Sims PC. Quantitative estimates of water tables and soil moisture in Holocene peatland from testate amoebae. The Holocene. 1998; 8: 261-273.
44. Patterson RT, Dalby A, Kumar A, Henderson LA, Boudreau REA. Arcellaceans (thecamoebians) as indicators of land-use change: settlement history of the Swan Lake area, Ontario as a case studt. J Paleolimnol. 2002; 28: 297-316.
45. Reinhardt EG, Goodman BN, Boyce JI, Lopez G, van Hengstum PJ, Rink WJ, Mart Y, Raban A. The Tsunami of December 13, 115 A.D. and the destruction of Herod the Great’s Harbor at Caesarea Maritima, Israel. Geology. 2006; 34 (12): 1061-1064.
46. van Hengstum PJ, Reinhardt EG, Medioli FS, Gröcke DR, (2007) Exceptionally preserved Late Albian (Cretaceous) Arcellaceans (Thecamoebians) from the Dakota Formation near Lincoln, Nebraska, USA. J Forami Res. 2007; 37: 300-308.
47. Medioli FS, Scott, DB, Collins ES, Mccarthy, FMG. Fossil thecamoebians: present status and prospects for the future. Hemleben C, Kaminski MA, Kuhnt W, Scott DB, editors. Proceedings of the NATO Advanced Study Institute on Paleoecology, Biostratigraphy, Paleoceanography and Taxonomy of Agglutinated Foraminifera: NATOASI Series, Series C: Mathematical and Physical Sciences 327: D. Reidel Publishing Company, Dordrecht-Boston.1990b; pp. 813-839.
48. Schönborn W, Dorfelt H, Foissner W, Krienitz L, Schäfer U. A fossilized microcenosis in Triassic amber. J. Euk Microbiol. 1999; 46: 571-584.
49. Schmidt AR, Schönborn W, Schäfer U. Diverse fossil Amoebae in German Mesozoic Amber. Palaeontology. 2004; 47: 185-197.
50. Poinar GO, Waggoner BM and Bauer U, (1993) Terrestrial soft-bodied protists and other microorganisms in Triassic Amber. Science. 1993; 259: 222-224.
51. Bassi DE, Fugagnoli A, Posenato R and Scott DB. Testateamoebae from the Early Jurassic of the western Tethys, northeast Italy. Palaeontology. 2008; 51: 1335-1339.
52. Martin-González A, Wierzchos J, Gutiérrez JC, Alonso J and Ascaso C. Microbial Cretaceous park: biodiversity of microbial fossils entrapped in amber. Naturwissenschaften. 2009; 96: 551-564.
53. Schmidt AR, von Eynatten H and Agreich M. The Mesozoic amber of Schliersee (southern Germany) is Cretaceous in age. Cretaceous Res. 2001; 22: 55-62.
54. Waggoner BM. The first fossil cyphoderiid testate amoebain Dominican Republic amber (Eocene–Oligocene). Paleobios. 1996b; 17: 17-19.
55. Foissner W, Schiller W. Stable for 15 million years: scanning electron microscope investigation of Miocene euglyphid thecamoebians from Germany, with description of new genus. Scutiglypha. Eur J Protistol. 2001; 37: 167-180.
56. Wolf M. Verkieste Amoeben in Steinkohlen aus dem Ruhrge-biet; erster Nachweis von Arcella Ehrenberg im Palaeozoikum [Pyritized amoebae in bituminous coals of the Ruhr District; first evidence of Arcella in the Paleozoic]. Paläontol Z. 1995; 69: 1-6.
57. Waggoner BM. Bacteria and protists from Middle Creta-ceous amber of Ellsworth County, Kansas. Paleobios. 1996a; 17: 20–26.
58. Boeuf O and Gilbert D. Présence de Thécamoebiens dugenre Trinema au Pliocène supérieur, découverte à Chilhac (Haute-Loire, France). C R Hebd Seances Acad Sci. 1997; 325: 623-627.
59. Bradley WH. Origin and the microfossils of the oil shale of the Green River Formation of Colorado and Utah. United States Geological Survey Professional Paper. 1931; 168: pp. 58.
60. Frenguelli G. Tecamebiani e Biatomee nel Miocene delNeuquen (Patagonia Settentrionale). B Uni Matemat Ital. 1933; 52: 33-43.
61. Kövàry J. Thekamöbak (Testaceak) a magyarorszagyalsopannoniai koru üled ekekböl. Földtani Közlöny. 1956; 86: 266–273.
62. Schiller W. Kieselige Thekamöben aus der Miozänen Kiesel-gur von Beuern/Vogelsberg im Vergleich mit rezentem Materialvon Borneo (Malaysia). Cour For Sekenbg. 1997; 201: 385-392.
63. Schiller W. Kieselige mikrofossilien aus demUnter-Oligozän von Sieblos/Rhön. Geologische Abhandlungen Hessen. 1998; 104: 173–199.
64. Schiller W. Kieselige Thekamöben aus del Mittel-Eozän desEckfelder Maares in der Eifel. Mainzer Naturwissenschaftliches Archiv. 1999; 37: 55–62.
65. Porter SA, Knoll A. Testate amoebae in the Neoproterozoic Era: evidence from vase-shaped microfossils in the Chuar Group, Grand Canyon. Paleobiology. 2000; 26: 360-385.
66. Kumar A, Farooqui A, Jha N. Early Permian glacio-marine thecamoebian assemblages from the northwest Himalayas, India. J Micropalaeontol. 2011; 30: 75-89.
67. Medioli FS, Scott, DB, Collins ES, McCarthy, FMG. Thecamoebians from the early Cretaceous deposits of Ruby Creek, Alberta (Canada). Hemleben C, Kaminski MA, Kuhnt W, Scott DB, editors. Proceedings of the NATO Advanced Study Institute on Paleoecology, Biostratigraphy, Paleoceanography and Taxonomy of Agglutinated Foraminifera: NATO ASI Series, Series C. Mathematical and Physical Sciences. 327: D. Reidel Publishing Company, Dordrecht-Boston, 1990a; pp. 793-812.
68. Farooqui A, Aggarwal N, Jha N. Thecamoebians from Late Permian Gondwana sediments of peninsular India. Eur J Protistol. 2014; 50: 89-105.
69. Wightman WG, Scott DS, Medioli FS and Gibling MR. Agglutinated foraminifera and thecamoebians from the late Carboniferous Sydney coalfield, Nova Scotia: paleoecology, paleoenvironments and paleogeographical implications. Palaeogeog Palaeoclimatol Palaeoecol. 1994; 106: 187-202.
70. Porter S, Meisterfeld R, Knoll A. Vase-shaped microfossils from the Neoproterozoic Char Group, Grand Canyon: a classification guided by modern testate amoebae. J Palaeontol. 2003; 77: 409-429.
71. Loeblich AR, Tappan H. Protista 2. Sarcodina chiefly ‘Thecamoebians’ and Foraminiferida, Part C. Moore RC, editor. Treatise on Invertebrate Palaeontology 2, Geological Society of America and University Kansas Press, Lawrence, Kansas. 1964.
72. Bhat GM and Bhat GD. Stratigraphy and depositional environments of Late Permian carbonates, Kashmir Himalaya. Wijayananda NP, Cooray PG, Mosley P, editors, Geology in South Asia-II, Geological Survey and mines bureau, Sri Lanka, Professional paper. 1997; 7: 205-223.
73. Shen SZ, Cao CQ, Henderson CM, Wang XD, Shi GR, Wang Y, et al. End-Permian mass extinction pattern in the northern peri-Gondwana region. Palaeoworld. 2006; 15: 3-30.
74. Algeo T, Hannigan R, Rowe H, Brookfield ME, Baud A, Krystyn L, et al. Sequencing events across the Permian-Triassic boundary, Guryul Ravine (Kashmir, India). Palaeogeog Palaeoclimatol Palaeoecol. 2007; 252: 328-346.
75. Proemse BC, Grasby SE, Wieser ME, Mayer B, Beauchamp B, Molybdenum isotopic evidence for oxic marine conditions during the latest Permian extinction. Geology. 2013; 41: 967-970.
76. Algeo TJ, Henderson CM, Tong J, Feng Q, Yin H, Tyson RV. Plankton and productivity during the Permian–Triassic boundary crisis: An analysis of organic carbon fluxes. Global and Planetary Change. 2013;105: 52–67.
77. Algeo TJ, Shen Y, Zhang T, Lyons TW, Bates SM, Rowe H, et al. Association of 34S-depleted pyrite layers with negative carbonate ?13C excursions at the Permian/Triassic boundary: evidence for upwelling of sulfidic deep-ocean watermasses. Geochemistry, Geophysics, Geosystems 9. 2008; Q04025 10 pp.
78. Medioli FS, Scott DB, Abbot BH. A case study of protozoan interclonal variability: taxonomic implications. J Foramin Res. 1987; 17: 28-47.
79. Medioli FS, Scott DB, Collins E, Asioli A, Reinhardt A. The thecamoebian bibliography. Palaeontol Electron. 1999; 3: 1-161. (http://palaeo- electronica.org/1999_1/biblio/issue1_99.htm).
80. Medioli FS, Bonnet L, Scott DB, Medioli B. The thecamoebian bibliography (2nd edn). Palaeontol Electron. 2003; 6: 1-107. (http://palaeo- electronica.org/2003_1/biblio/issue1_03.htm).
81. Smirnov A, Chao E, Nassonova E, Cavalier-Smith T. A Revised Classification of Naked Lobose Amoebae (Amoebozoa: Lobosa). Protist. 2011; 162: 545-570.
82. Ogden CG, Hedley RH. An atlas of freshwater testate amoebae. Oxford University Press, London, UK, 1980.
83. Cavalier-Smith T. A revised six-kingdom system of life. Biol Rev. 1998; 73: 203-266.
84. Carpenter W B. On the systematic arrangement of the Rhizopoda. Natural History Review. 1861; 1: 456-472.
85. Cavalier-Smith T. Megaphylogeny, cell body plans, adaptive zones: causes and timing of eukaryote basal radiations. J Eukaryot Microbiol. 2009; 56: 26-33.
86. Smirnov AV, Nassonova ES, Berney C, Fahrni J, Bolivar I, Pawloski J.  Molecular phylogeny and classification of the lobose amoebae. Protist; 2005; 156: 129-142.
87. Cavalier-Smith T, Chao EE, Oates B. Molecular phylogeny of Amoebozoa and evolutionary significance of the unikont Phalansterium. Eur J Protistol. 2004; 40: 21-48.
88. Kent WS, (1880–81) A Manual of the Infusoria: Including a Description of All Known Flagellate, Ciliate, and Tentaculiferous Protozoa, British and Foreign, and an Account of the Organization and Affinities of the Sponges. D. Bogue. London. 1880-81; pp. 472.
89. Ehrenberg CG. Über die Entwickelung und Lebensdauer der Infusionsthiere, nebst ferneren Beiträgen zu einer Vergleichung ihrer organischen Systeme. Königliche Akademie der Wissenschaften zu Berlin. Physikalische Abhandlungen (1831). 1832; pp. 1-154
90. Jung W. Südchilenische Thekamöben (Aus demsüdchilenischen Küstengebiet, Beitrag 10). Arch Protistenk. 1942; 95: 253-356.
91. Stein SFN von. Über die ihm aus eigener Untersuchung bekannt gewordenen Süswasser-Rhizopoden. Königliche Böhmische Gesellschaft der Wissenschaften Abhandlungen Ser 5. Berichte der Sectionen, 1859; 10: 41-43.
92. Ehrenberg, C.G. Die Infusionsthierchen als vollkommeneOrganismen. Ein Blick in das tiefere organische Leben der Natur. L. Voss, Leipzig, 1838; 2: pp. 1–547.
93. Foissner W, Korganova GA. The Centropyxis aerophila complex (Protozoa: Testacea). Acta Protozool. 2000; 39: 257–273.
94. Ehrenberg CG. Über noch jetzt zahlreich lebende Thier-arten der Kreidebildung und den Organismus der Polythalamien. Abhandl. Königl. Akad. Wiss. Berlin 1839. 1841; pp. 81– 174.
95. Schulze FE. Rhizopodenstudien VI. Arch Mikroskop Anatom. 1877; 13: 9–30.
96. Penard E. Faune Rhizopodique Du Basin Du Léman. Henry Kündig. Genève. 1902; pp. 714.
97. Carter HJ. Notes on the fresh water Infusoria of the island of Bombay. Ann Mag Nat Hist. Ser 2. 1856; 18: 221–249.
98. Deflandre G. Le genre *Centropyxis* Stein. Arch Protistenkd. 1929; 67, 322–375.
99. Vasicek M, Ruzicka B. Namurian Techamoebina from the Ostraxa-Karxina coal district. Sbornik Naradniho Museav Praze, Rada B, Prirodni Vedy Acta Musei Nationalis Pragae, Ser. B. Historia Naturalis. 1957; 13: 333–340.
100. Jennings HS. Heredity, variation and the results of selection in the uniparental reproduction of Difflugia corona. Genetics 1. 1916; 407-534.
101. Medioli FS, Scott DB. (1983) Holocene Arcellacea (Thecamoebians) from eastern Canada. Cushman Foundation For Foraminiferal Research Special Publication 21.1983; pp. 63.
102. Kumar A, Dalby AP. Identification key for Holocene lacustrine Arcellacean (thecamoebian) taxa. Palaeontol Electron. 1998; 1: 1–39.
103. Charman DJ, Hendon D, Woodland W. The identification of peatland testate amoebae. Quaternary Research Association Technical Guide. 9, London; 2000. pp.147.

S1 Permission from Elsevier [Elsevier], under a CC BY license (3606870471810) original copyright year- 2006)

S2 Written permission from the original author.

**Figure Captions**

**Figure 1:** Palaeoposition of the Guryul Ravine Section in the northern peri-gondwanan region during the P-T transition (after Shen et al., 2006; base map after Ziegler et al., 1997 under a CC BY license (3606870471810); with permission from [Elsevier], original copyright year- 2006)

**Figure 2**: Permian Triassic Boundary (PTB) study section at Guryul Ravine, Kashmir (after Kapoor 1996) Red Triangles pointing to the levels of reported occurrence of thecamoebians along with previously studied faunal elements. Conodont biozones after Algeo et al., 2007. PTB- Permian-Triassic boundary; LPME- Late Permian Mass Extinction. Occurrence of thecamoebians

**Figure 3:** Thecamoebian fossil record of the families Arcellidae and Centropyxidae Thecamoebians preserved in sediments noted by a circle, whereas thecamoebians preserved in amber are noted by a triangle. The basal part of the figure depicting ecology of the genera *Centropyxis* and *Arcella* through time has been modified in the light of this study and other published data. (a) Wolf, 1995; (b) Kumar et al., 2011; (c) Farooqui et al., 2014; (d) Wightmann et al., 1994; (e) Medioli et al., 1990a; (f) Schönborn et al., 1999; (g) Poinar et al., 1993; (h) Schmidt et al., 2004;(i) Waggoner, 1996b; (j) van Hengstum et al., 2007; (k) Porter and Knoll, 2000 and (*) denotes this study. The geological time axis is not to scale. The figure has been modified after van Hengstum et al. 2007 (with written permission from the original author van Hengstum).

**Figure 4:** Location and geological map of the study area also depicting geological section of Guryul Ravine and the recovered thecamoebians (modified after Bhat and Bhat, 1997).

**Figure 5:** Relative abundance of recovered thecamoebians showing high diversity within the genera Centropyxis. Unit E2 displays maximum diversity. The scale represents number of forms present.

**Figure 6:** A,B. *Centropyxis platystoma* (Penard, 1890) Elongated shell, apertural view, 3- shows inner linning of the test, slide no. 14974.

C. *Cucurbitella tricuspis* (Y53/4), slide no.14974.

D. *Centropyxis* Stein, 1857, (Q40/3), test agglutinated and small in size, silde no.14881.

E-G. *Centropyxis* *aerophila*-complex Foissner and Korganova, 2000 (E,F: S11/4; 7: E35/4), E,F- apertural and dorsal view, respectively, G- apertural view, slide nos. (E,F) 14976, (G) 14973.

H,I. *Cyclopyxis eurystoma* (Deflandre, 1929) (R15/3) H-Ventral view and I-dorsal view, test is small in size, slide no. 14976. J. Arcella Ehrenberg, 1832 (T63/4), slide no.14971.

K,L. Incertae sedis (U18/2), test shows two apertures, K-aperture one, L- aperture two,

test small in size, slide no. 14976.

**Figure 7:** A. *Centropyxis aculeata* var. *spinosa* Ehrenberg, 1832 (Q31/4), apertural view, slide no. 14974.

B. *Centropyxis aculeata* var. *spinosa* Ehrenberg, 1832 (E41/3), B-dorsal view showing spines, xenosomes on the test, C. ventral/apertural view, slide no. 14869

D. *Centropyxis aculeata* var. *spinosa* Ehrenberg, 1832 (T40/3), apertural view showing 9-10 spines at the margin, degraded brown organic matter and detrital grains on the agglutinated test, slide no. 14975.

E. *Centropyxis aculeata* var. *spinosa* Ehrenberg, 1832 (U21/3), apertural view, dark coloured test with three spines, slide no. 14976

F. *Centropyxis aculeata* var. *spinosa* Ehrenberg, 1832 (N18/4), apertural view, test light coloured agglutinated formed by material from the surrounding, slide no. 14973

G, H. *Centropyxis aculeata* var. *discoides* Penard, 1902 (P29/2), test constituted of both autosomes and xenosomes, slide no. 14974.

I. *Centropyxis aculeata* var. *discoides* Penard, 1902 (K34/1), slide no. 14976.

J-L *Centropyxis aculeata* var. *discoides* Ehrenberg, 1832 (H30/4), apertural view, J, image is focussed on the margin of the test showing two broken spines, K- aperture focused, L- Margin focused showing a complete spine, slide no. 14976.

**Figure 8:** (A, B) *Centropyxis aculeata* var. *spinosa*, (H20/2), A- apertural view (lobate aperture), B- Dorsal view showing spines on the folded dorsal surface, slide no. 14976. C, D. *Centropyxis aculeata* (R36/3), C- dorsal view showing broken spines at the margin, D- apertural view shows ventral margin of the aperture connected to the dorsal face by bridges (shown by arrows) slide no.14869.

E-L, *Centropyxis aculeata* (E,F: Q22/3; G,H: P31/4; I,J: R41/2; K,L: N18/2) (E,G,I, K apertural views; F, H, J, L dorsal views showing spines), test covered with sand grains and BDOM from the vicinity, sand grains impart grey colour to the tests, slide nos.(E,F) 14975, (G,H) 14869, (I,J) 14975, (K,L) 14972. Scale bar: 20 µm.

**Table 1:** A list of examined thecamoebian forms and their morphological details. The morphological features (hypostome shape and dimensions, number of visible spines) have been subjected to post-depositional compression and compaction and are thus incomparable to the recent forms. All the morphometric measurements are in µm.

| **Examined Specimen (Position in the figs.)** | **Shell Length** | **Maximum Shell width** | **Pseudostome length of long axis, short axis** | **Spines** |
| --- | --- | --- | --- | --- |
| 1. *Centropyxis. aculeata var. spinosa* (7 A) | 90 | 90 | 33 | 4 visible |
| 2. *C. aculeata* var. *discoides* (7 J,K,L) | 104 | 100 | 44,33 | 3 visible: 1 complete, 2 broken |
| 3. *C. aculeata* var. *spinosa* (7 E) | 120 | 102 | 37 | 4 visible: 3 complete, 1 broken |
| 4. *C. aculeata var. spinosa* (8 A,B) | 117 | 120 | 50,29 | 3 visible: 1 not clear |
| 5. *C. aculeata* var. *spinosa* (7 B,C) | 90 | 85 | 36,30 | 3 visible |
| 6. *C. aculeata* var. *Spinosa* (7 D) | 140 | 147 | 64,50 | 9-10 visible |
| 7. *C. aculeata* var. *Spinosa* (7 F) | 130 | 120 | 45,32 | 4 visible |
| 8. *Cucurbitella tricuspis* (6 C) | 56 | 56 | 19 | Absent |
| 9. *C. aerophila* (6 E,F) | 60 | 60 | 25,20 | Absent |
| 10. *C. aerophila* (6 G) | 55 | 50 | 22,13 | Absent |
| 11. *Centropyxis platystoma* (6 A,B) | 87 | 45 | 30 | Absent |
| 12. *Centropyxis aculeata* (8 C,D) | 114 | 114 | 53,21 | 3 broken |
| 13 *C. aculeata* (8 G,H) | 85 | 85 | 26,19 | 4-5: Not clearly visible |
| 14. *C. aculeata* (8 E,F) | 120 | 109 | 40,30 | 4-5: |
| 15. *C. aculeata* (8 I,J) | 96 | 90 | 38,23 | 5 |
| 16. *C. aculeata* (8 K,L) | 138 | 98 | 47,50 | 4 |
| 17. *C. aculeata* var. *discoides* (7 G,H) | 112 | 109 | 40 | 1 visible |
| 19. *C. aculeata* var. *discoides* (7 I) | 111 | 120 | 46 | 1 broken |
| 20.*Cyclopyxis eurystoma* (6 H,I) | 37 | 37 | 19 | Not clearly visible |
| 21.*Centropyxis* Stein, 1857 (6 D) | 61 | 53 | 27,21 | 1 broken |
| 22. *Centropyxis* *sp.* | 54 | 54 | 20,14 | Absent |
| 23. *Arcella* (6 J) | 80 | 80 | 16 | Pores are visible around the aperture |
| 24. Unidentified | 37 | 37 | 2 apertures diameter 15, 20 |  |
